# Thyroid Cancer Incidence in New Jersey: Time Trend, Birth Cohort and Socioeconomic Status Analysis (1979–2006)

**DOI:** 10.1155/2011/850105

**Published:** 2011-11-30

**Authors:** Lisa M. Roche, Xiaoling Niu, Karen S. Pawlish, Kevin A. Henry

**Affiliations:** Cancer Epidemiology Services, New Jersey Department of Health and Senior Services, P.O. Box 369, Trenton, NJ 08625-0369, USA

## Abstract

The study's purpose was to investigate thyroid cancer incidence time trends, birth cohort effects, and association with socioeconomic status (SES) in New Jersey (NJ), a high incidence state, using NJ State Cancer Registry data. Thyroid cancer incidence rates in each sex, nearly all age groups, two major histologies and all stages significantly increased between 1979 and 2006. For each sex, age-specific incidence rates began greatly increasing in the 1924 birth cohort and, generally, the highest thyroid cancer incidence rate for each five-year age group occurred in the latest birth cohort and diagnosis period. Thyroid cancer incidence rates were significantly higher in NJ Census tracts with higher SES and in counties with a higher percentage of insured residents. These results support further investigation into the relationship between rising thyroid cancer incidence and increasing population exposure to medical (including diagnostic) radiation, as well as widespread use of more sensitive diagnostic techniques.

## 1. Introduction

While U.S. incidence rates for many common cancers decreased or remained stable between 1975 and 2006, thyroid cancer incidence increased over 125% [1]. For 2002–2006, New Jersey's (NJ) average annual age-adjusted rate was a third higher than the U.S. rate (12.8 versus 9.6 per 100,000) [1] and NJ ranked fifth among 47 states in 2001–2005 thyroid cancer incidence [2]. Although considered relatively uncommon, thyroid cancer currently ranks seventh in cancer incidence among U.S. and NJ women [1, 3].

The major risk factor for thyroid cancer is exposure to ionizing radiation, usually in childhood, either externally from therapeutic X-radiation [4, 5] or internally through treatment with radioactive iodine (^131^I) and possibly radioactive fallout (^131^I) from above-ground nuclear bomb testing in Nevada in the 1950s [4, 6–8]. An association between exposure to diagnostic X-radiation and thyroid cancer has been found in several case-control studies [9]. Other risk factors are a history of thyroid disease including nodules, goiter, and hyperthyroidism, familial or genetic factors and iodine deficiency or excess [4].

X-radiation treatment of children for benign conditions of the head and neck in the 1920s through the 1950s resulted in higher thyroid cancer incidence through the 1945 birth cohort [4, 10–12]. Increases in thyroid cancer incidence also are attributed to widespread use since the 1980s of more sensitive diagnostic techniques such as ultrasonography and fine-needle aspiration [13, 14]. However, these two factors do not fully explain the continuing large increases in thyroid cancer incidence in the past three decades [9, 15, 16].

Socioeconomic status (SES) is commonly believed to be an important determinant of health status in individuals and populations [17]. In the USA, low SES is associated with higher incidence of many cancer types, for example, lung, stomach, and cervical, while high SES is related to greater breast cancer and melanoma incidence [17]. Some studies have found positive associations between high levels of income, education, or other SES indicators and thyroid cancer [18–21], while other studies found a negative or no association [22–24]. Two recent studies show a positive correlation between health insurance coverage and thyroid cancer incidence [18, 19].

In recent years, U.S. researchers have used deprivation indices developed from several census variables as measures of SES, rather than single census variables such as income or education. These indices are commonly developed using data reduction techniques that condense a large number of variables into a smaller number of components to more accurately reflect the multidimensional character of community socioeconomic position [25]. Deprivation indices have been used to study the relationships between SES and cancer incidence and stage at diagnosis [26–28].

The purpose of this study was to investigate thyroid cancer incidence time trends and birth cohort effects from 1979 to 2006 in New Jersey (NJ), a high incidence state. We also investigated the relationship between thyroid cancer incidence and SES, as defined by a deprivation index and insurance status.

## 2. Methods

The New Jersey State Cancer Registry (NJSCR) is the population-based cancer incidence registry that serves the state of NJ, with a diverse population of over 8.7 million people. The NJSCR has participated in the Centers for Disease Control and Prevention's National Program of Cancer Registries since it began in 1994 and is a National Cancer Institute (NCI) Surveillance, Epidemiology, and End Results (SEERs) expansion registry. Reciprocal reporting agreements are maintained with Delaware, Florida, Maryland, New York, North Carolina, and Pennsylvania. The NJSCR includes patient demographic characteristics (birthdate, age, sex, race, ethnicity) and clinical information on each cancer diagnosis. The primary site and histology of each case are coded to the International Classification of Diseases for Oncology (ICD-O), third edition [29], and the stage at diagnosis is coded according to SEER summary stage [30, 31]. Each case's residential address at the time of diagnosis is geocoded and assigned a county and census tract. The North American Association of Central Cancer Registries awarded the Gold Standard, the highest possible, to the NJSCR for quality and completeness of 1995 through 2006 data.

### 2.1. Time Trends

Primary invasive thyroid cancer cases (ICD-O-3 site code 73.9) diagnosed in NJ residents during 1979–2006 were included, except cases reported only by death certificate or autopsy report. Directly age-adjusted thyroid cancer incidence rates per 100,000 population by year of diagnosis, sex, race, ethnicity, histology, stage at diagnosis, tumor size, and age-specific rates were calculated using 1979–2006 NJ population estimates from the National Center for Health Statistics (based on U.S. Census Bureau data) [32] and the 2000 U.S. Standard Population. The time trends for the race category Asian and Pacific Islanders were not calculated due to low numbers. U.S. thyroid cancer incidence rates by year of diagnosis and sex were calculated with data from nine SEER registries using the same methods as for NJ rates. All rates were calculated in NCI's SEER*Stat software [33].

Joinpoint regression software from NCI [34] was used to calculate the average annual percent changes (AAPCs) in NJ thyroid cancer incidence rates by sex, race, age, histology and stage, and in U.S. rates by sex during 1979–2006 and the most recent ten years, 1997–2006. AAPCs also were calculated by Hispanic ethnicity but only for 1997–2006, as accurate data are not available before 1990. Statistical significance of the AAPCs was determined with 95% confidence intervals. AAPCs were not calculated by tumor size because the data were incomplete before 1999 and the percentages of cancers with unknown size were greater than 20% for 1999–2003.

The AAPC is a summary measure of a time trend over a prespecified fixed period, for example, 1979–2006, and is computed as a weighted average of the annual percent changes (APCs) from the best underlying Joinpoint regression model, with weights equal to the intervals (line segments) between joinpoints [35]. To obtain the best underlying Joinpoint regression model, the software identifies points (joinpoints), where the rate of change in trend data (e.g., annual cancer incidence rates) significantly increases or decreases, and provides an estimated APC between each joinpoint [36]. It starts with zero joinpoints and continues to add joinpoints up to a set maximum until adding one more joinpoint does not statistically significantly improve the model.

### 2.2. Birth Cohort Analysis

For the birth cohort analysis, thyroid cancer cases younger than 20 were excluded due to the small number of cases, and cases aged 85 and older were excluded because birth cohorts could not be assigned. Age-specific incidence rates by five-year grouped diagnosis year and nine-year overlapping birth cohort were calculated for each sex using SEER*Stat. The 13 age groups were 20–24, 25–29…80–84, and the six five-year diagnosis periods were 1979–83, 1984–88,…,2004–06. The last diagnosis period includes only three years as 2006 was the last year of complete data. Nine-year overlapping birth cohorts (except the last birth cohort was 7 years) were derived using Cohort = Period − Age, yielding a total of 18 birth cohorts from 1895–1903 to 1980–1986 [37]. The cohorts are identified by the mid-year of birth, for example, the 1900–1908 birth cohort is labeled “1904”.

### 2.3. Socioeconomic Status (SES) and Health Insurance

The relationship between NJ thyroid cancer incidence and SES was examined for the five years (1998–2002) centered at the year 2000, the most recent year with publicly available U.S. Census data at the census tract level. Since the NJSCR does not collect individual case information on SES measures such as income or educational attainment, a standardized deprivation index, similar to one used in other studies [26], was developed with 2000 U.S. Census data for NJ using principle component analysis [38] and SAS (SAS procedure PRINCOMP [39], SAS statistical software, SAS Institute Inc., Cary, NC). Of the 1,950 census tracts in NJ, 23 with missing SES variables were excluded from the development of the deprivation index. The first component scores for each census tract were calculated using eleven census variables—percent families below the poverty line, median household income, percent female—headed households, percent adults with less than high school education, percent males and females in management jobs, percent males and females in professional jobs, percent unemployment, percent renters, and percent owner-occupied homes worth less than $300,000. The 1,927 census tracts were grouped into SES quartiles based on the deprivation index scores; the higher the deprivation index score, the more deprived the tract. Age-adjusted thyroid cancer incidence rates per 100,000 population by sex for each SES quartile were calculated using U.S. Census Bureau 2000 population data [32] and the 2000 U.S. Standard Population. For the rate ratios, the highest SES quartile was the referent for the other three; the significance level was set at *P* < 0.05 [40]. The rates and rate ratios were calculated in SEER*Stat. This analysis was not done by race because the multiple race categories in the 2000 Census were not allocated to single race categories at the census tract level.

Estimates by the U.S. Census Bureau of the percentage of residents aged 0–64 covered by health insurance in 2005 and 2006 were averaged for each NJ county [41, 42]. County thyroid cancer age-adjusted incidence rates among persons 0–64 were calculated for 2005 and 2006 in SEER*Stat using 2005-2006 NJ population estimates from the National Center for Health Statistics (based on U.S. Census Bureau data) [32] and the 2000 U.S. Standard Population. A Pearson correlation coefficient between the 21 county thyroid cancer incidence rates and average health insurance percent coverage weighted by county populations was calculated using SAS.

## 3. Results

### 3.1. Time Trends and Birth Cohorts

After exclusion of the death certificate only (*n* = 116) and autopsy only (*n* = 45) cases, 15,576 invasive primary thyroid cancer cases diagnosed between 1979 and 2006 were analyzed. More than 98% of the included cases were histologically confirmed. The annual count ranged from 229 cases in 1981 to 1,316 cases in 2006. Three-fourths of the cases were female, 86% white, 54% younger than 50 at diagnosis, 76% papillary histology, and 63% diagnosed at the local stage.

Overall, NJ thyroid cancer AAPCs showed significant incidence rate increases between 1979 and 2006, accelerating in the most recent ten years (Table 1). The 1979–2006 rate increases were more prominent among NJ women than men and were greatest for black women, followed by white women, black men, and white men. Rates significantly increased in each age group except women 80–84, with the steepest increases in both sexes combined 45–49 and 70–74 and women 35–39 and 70–74. The greatest increase occurred in thyroid cancer of papillary histology, although follicular thyroid cancer also significantly increased. Thyroid cancer diagnosed at the local, regional, and distant stages significantly increased, while unknown stage significantly decreased (note: these AAPCs should be interpreted with caution as the percentage of unknown stage was over 10% for most years 1979–1995). Female Hispanic rates significantly increased in 1997–2006, while male Hispanic rates did not. From 1999 to 2006, incidence rates increased for each tumor size except unknown size decreased: ≤1 cm–1.4 to 4.6; 1.1–2.0 cm–1.6 to 4.1; 2.1–5.0 cm–2.2 to 4.2; >5.0 cm–0.5 to 0.7 (data not shown). NJ AAPCs were higher than U.S. AAPCs in both time periods for total, males and females, for example, 1979–2006—5.4%, 4.9%, 6.0% versus 3.8%, 3.3%, 3.8%, respectively (U.S. data not shown in Table 1).

**Figure 1:**
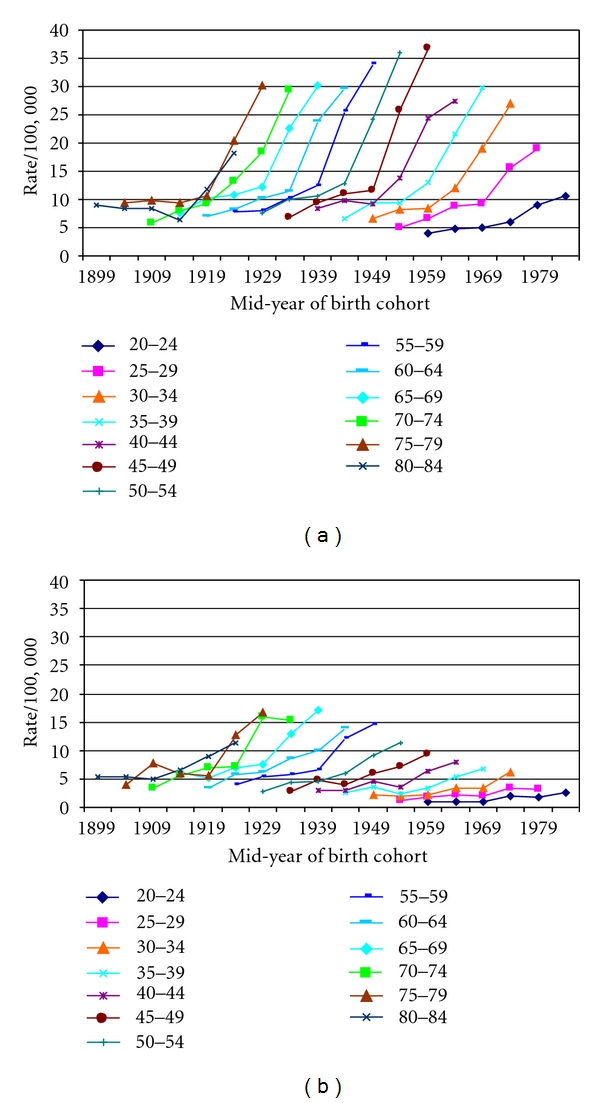
(a) Female thyroid cancer incidence rate by age and birth cohort, New Jersey, 1979–2006, *N* = 11, 215. (b) Male thyroid cancer incidence rate by age and birth cohort, New Jersey, 1979–2006, *N* = 3, 772.

Age-specific thyroid cancer incidence per 100,000 NJ women greatly increased beginning with the two oldest age groups in the 1924 birth cohort and continued to rise in younger age groups, including the 20–24 age group, as well as in the older age groups in subsequent birth cohorts (Table 2(a), Figure 1(a)). Every age group experienced steep rate increases with increasingly later birth cohorts and diagnosis periods. The highest rates occurred among women in the 45–49, 50–54, and 55–59 age groups from the 1959, 1954, and 1949 birth cohorts. Male age-specific thyroid cancer incidence per 100,000 also increased greatly beginning with the two oldest age groups in the 1924 birth cohort (Table 2(b), Figure 1(b)). As with women, for each age group the rates in the most recent two birth cohorts generally were much higher than for the earlier birth cohorts, but the highest rates occurred in older men in earlier cohorts, aged 65–69, 70–74, and 75–79 of the 1939, 1934, and 1929 birth cohorts.

### 3.2. Socioeconomic Status (SES) and Health Insurance

Of the 3,924 thyroid cancer cases included in the SES deprivation index analysis, approximately 33% and 14% were in the highest and lowest SES quartiles, respectively (Table 3). The highest SES quartile had the highest thyroid cancer incidence rate—10.6 per 100,000 population, and each subsequently lower SES quartile had a lower rate—6.5 for the lowest SES quartile. The rates for the two lowest SES quartiles were significantly lower than the rate in the highest SES quartile (*P* < 0.05) while there was no statistical difference between the two highest SES quartiles; the same pattern held for each sex. Although female rates were much higher than male rates, the rate ratios were similar for the two sexes.

In NJ's 21 counties, the average percent of residents ages 0–64 with health insurance in 2005-2006 ranged from 73% to 89% and the 2005-2006 age-adjusted thyroid cancer incidence rate (*n* = 2, 026) ranged from 8.6 to 16.7 per 100,000 population. There was a significant positive correlation between the counties' percent health insurance coverage and incidence rates (Pearson correlation coefficient = 0.56, *R* ^2^ = 0.32, *P* = 0,008). Thus, approximately a third of the variation in county thyroid cancer incidence rates can be explained by the percent of residents with health insurance.

## 4. Discussion

### 4.1. Time Trends and Birth Cohorts

Thyroid cancer incidence in NJ men and women increased substantially from 1979 to 2006, most notably in the past ten years. This time trend mirrored that in the USA; however, NJ AAPCs and incidence rates after 1999 were substantially higher than the USA's. Our birth cohort analysis showed that thyroid cancer age-specific incidence rates began greatly increasing in the oldest members of the 1924 birth cohort, generally with the highest thyroid cancer incidence rate for each five-year age group occurring in the latest birth cohort and diagnosis period for which data were available.

Zheng et al. found that the increase in thyroid cancer incidence in Connecticut between 1935 and 1992 occurred in the 1915–1945 birth cohorts, with decreasing incidence in subsequent cohorts [10]. The authors noted that these results were consistent with the hypothesis that radiation treatment in the 1920s–1950s of children with benign head and neck conditions was largely responsible for the rise in thyroid cancer incidence rates in birth cohorts up to 1945. However, a more recent age-period-cohort modeling of U.S. SEER papillary thyroid cancer data from 1973 through 2004 found a strong continuous birth cohort effect, as well as a period effect [9]. The researchers concluded that the period effect, likely due to increased diagnosis of thyroid cancer, explains some of the rise in thyroid cancer rates, and that birth cohort-related increased exposure to environmental factors such as diagnostic X-rays and hormone disrupters (polybrominated diphenyl ethers, other polyhalogenated aromatic hydrocarbons) also may have contributed. The results of our birth cohort analysis are consistent with those from the more recent study [9]. What could account for the continuing increase in thyroid cancer incidence, specifically in birth cohorts after 1944?

More sensitive diagnostic techniques for thyroid cancer, widely used since the 1980s (ultrasound) and 1990s (fine-needle aspiration), increased the ability to diagnose very small thyroid cancers that would have been missed previously [14]; however, recent research indicates that this does not explain all the increase [9, 15, 16]. Tumors of all sizes and stages have increased, not just small and early-stage tumors, and thyroid cancer mortality rates have not changed [9, 15, 16]. Also, no plateau in thyroid cancer incidence rates has occurred, as expected after a temporary increase in rates due to new, more sensitive diagnostic techniques becoming widely used.

Assuming that part of the rise in thyroid cancer incidence rates reflects an actual increase in thyroid cancer, reasons for the increase related to thyroid cancer etiology should be explored. U.S. population exposure to ionizing radiation, the major known risk factor for thyroid cancer, has steadily increased in the past few decades. The National Council on Radiation Protection and Measurements (NCRP) estimates that the effective radiation dose to the U.S. population in 2006 was an average of 6.2 milli-Sieverts (mSv) per person [43]. Most of the average dose per person was from ubiquitous background radiation (3.1 mSv) and medical radiation excluding radiotherapy (3.0 mSv). While background radiation increased very little since 1982, the last year for which the NCRP previously published radiation exposure data for the U.S. population, the average dose per person from medical exposure increased from 0.54 mSv in 1982 to 3.0 mSv in 2006 [43, 44]. This increase was primarily due to increases in the use of computed tomography (CT, about 1.5 mSV average dose per person in 2006), interventional fluoroscopy, and nuclear medicine—all relatively high-dose procedures [44], higher than standard diagnostic X-rays [45]. The number of CT procedures per year in the USA increased over 10% a year between 1993 and 2006, from 18.3 million to 62 million [44].

As noted by NCRP, not everyone in the USA receives the average medical radiation dose. Women, elderly, and sick individuals are more likely to receive higher doses [43, 44], confirmed by a recent study of exposure to ionizing radiation from medical imaging procedures received in 2005–2007 by more than 650.000 adult (18–64 years old) enrollees in a very large health care organization [46]. The authors estimated an annual mean effective dose of 2.4 ± 6.0 mSv per enrollee with a wide distribution of dose: 19.4% received >3–20 mSv; 1.9% >20–50 mSv and 0.2% >50 mSv. Women and older adults had higher effective doses, although 30% of men and 40% of women who received doses greater than 20 mSv per year were between 18 and 49 years of age.

The Board on Radiation Effects Research (BRER), National Academy of Sciences, recently concluded that the linear no-threshold risk model applies to low-level ionizing radiation (including X-radiation and gamma radiation) and cancer, with the risk of solid cancers in organs rising proportionately with cumulative exposure [47]. This risk model predicts that about one of 100 people exposed to 0.1 Sv (100 mSV) would likely develop a solid cancer or leukemia. The preferred model of excess relative risk (ERR) for thyroid cancer is sex-specific with a factor that doubles the risk for women compared to men. A recent analysis using information from the NCRP report [43, 44] and BRER risk models [47] estimated that about 1,000 thyroid cancers will result from CT scans performed in 2007 [48].

Although childhood exposure to ionizing radiation has been considered mainly responsible for increased thyroid cancer risk due to radiation [4], two recent studies of Japanese atomic bomb survivors found an association between exposure of adult women to ionizing radiation and thyroid cancer [49, 50].

Environmental chemicals that have been associated with disruption of thyroid function and/or with thyroid cancer—dioxins and polyhalogenated aromatic hydrocarbons (e.g., polybrominated diphenyl ethers, polychlorinated biphenyls)—are suspected to account for some of the increase in thyroid cancer incidence [9, 16]. Obesity has been implicated in thyroid cancer etiology, perhaps by increasing thyroid-stimulating hormones; thus increasing obesity prevalence in the USA may have contributed to the rise in thyroid cancer incidence [16, 51]. Changes in various reproductive and hormonal factors may be partial explanations for the increase in thyroid cancer incidence rates, especially since thyroid cancer continues to affect women much more than men and peaks at an earlier age in women, but current evidence is weak and inconsistent [4, 51]. Increased dietary iodine intake also has been suggested as a possible factor in the increasing incidence of thyroid cancer [15]; however, changes in iodine intake affect specific histologic types differently and the net effect on total thyroid cancer incidence may be minimal [4].

### 4.2. Socioeconomic Status (SES) and Health Insurance

The results of previous research on thyroid cancer and SES, most using single measures of SES, have been mixed [18–24]. One recent study examining 1995–2004 thyroid cancer incidence in Wisconsin counties found a moderate positive correlation between thyroid cancer incidence and median household income and percentage of residents with a college degree [18]. Another study using 1973–2003 data from seventeen SEER registries found that thyroid cancer patients were more likely to reside in ZIP codes with higher median income [19]. Two earlier studies, one of a cohort from a large health plan in the San Francisco Bay Area [20] and another using Los Angeles cancer registry data [21], found positive relationships between thyroid cancer and high educational attainment [20] and an SES indicator based on U.S. Census data on household income and education at the census tract level [21]. Although we used a complex deprivation index for SES and different statistical techniques, our results were similar—higher SES was associated with higher thyroid cancer incidence rates.

A case-control study of Southeast Asian women living in the San Francisco Bay Area found that lower educational attainment was associated with an increased risk of thyroid cancer [22] and two case-control studies from the 1980s, in Hawaii [23] and Connecticut [24], found no association between thyroid cancer and education or occupation. These contradictory results suggest that the positive relationship between SES and thyroid cancer may be reversed for certain populations, for example, Asian and Pacific Islanders, and may be a more recent occurrence.

The authors of the Wisconsin and Los Angeles cancer registry studies noted that the positive association between thyroid cancer and SES may be due to increased diagnosis due to better access to health care in higher SES groups [18, 21]. However, in the San Francisco cohort study, the positive association of thyroid cancer with a high educational level was not believed to be due to detection bias as everyone in the cohort had equal access to health care [20].

The Wisconsin county study found a strong positive correlation between thyroid cancer incidence and percent of residents covered by health insurance [18], and the SEER study found positive correlations between state and zip code level health insurance status and thyroid cancer [19]. The Pearson correlation coefficients in these two studies were similar to ours, *r* = 0.41 and 0.56, respectively. This correlation is consistent with the findings of a positive association between high SES and thyroid cancer, as persons of higher SES are more likely to have health insurance [52].

The results of our study and others showing a positive association between thyroid cancer and high SES and health insurance coverage also are consistent with the hypothesis that increased exposure to medical (including diagnostic) ionizing radiation explains part of the rise in thyroid cancer.

In conclusion, thyroid cancer incidence increased greatly in NJ between 1979 and 2006, especially since 1997, with no indication of stabilizing. Our time trend, birth cohort, and SES results support further investigation into the relationship between rising thyroid cancer incidence and increasing population exposure to medical (including diagnostic) radiation, as well as widespread use of more sensitive diagnostic techniques. In the meantime, measures to encourage appropriate use of medical radiation would be prudent.

## Figures and Tables

**Table 1:** Thyroid cancer incidence trends in New Jersey by sex, race, ethnicity, and age. Histology and stage, 1979–2006, *N* = 15, 576^a^.

		Cases 1979–2006	Rate^b^ 1979–2006	AAPC^c^ 1979–2006	AAPC^c^ 1997–2006
Total		15,576	6.9	5.4*	10.3*
Sex	Male	3,899	3.7	4.9*	8.2*
	Female	11,677	9.9	6.0*	10.9*
Race					
Male	White	3,439	3.9	5.0*	8.4*
	Black	239	2.1	5.6*	5.6*
	API^d^	110^d^	4.5^d^	—	12.5*
Female	White	10,014	10.3	6.1*	10.0*
	Black	870	7.3	7.2*	11.4*
	API^d^	396^d^	13.7^d^	—	10.5*
Ethnicity^d^					
Male	Hispanic^d^	169^d^	3.8^d^	—	2.0
	Non-Hispanic^d^	2,037^d^	5.6^d^	—	8.1*
Female	Hispanic^d^	753^d^	14.2^d^	—	7.4*
	Non-Hispanic^d^	6,213^d^	15.5^d^	—	12.2*
Age	0–19	362	0.6	3.6*	3.6*
	20–24	578	3.7	4.2*	4.2*
	25–29	989	6.0	5.2*	5.2*
	30–34	1,402	7.8	5.4*	9.0*
	35–39	1,651	9.2	5.7*	10.7*
	40–44	1,719	10.4	4.8*	8.2*
	45–49	1,699	11.7	6.3*	11.2*
	50–54	1,526	11.8	6.0*	9.7*
	55–59	1,393	12.1	6.1*	10.1*
	60–64	1,105	10.9	6.0*	9.4*
	65–69	1,039	11.8	5.6*	10.8*
	70–74	835	11.2	6.7*	6.7*
	75–79	703	12.2	4.8*	12.4*
	80–84	348	9.1	2.8*	10.5*
	85+	227	7.4	2.6*	2.6*
Female	0–19	283	0.9	3.4*	3.4*
	20–24	471	6.1	4.2*	4.2*
	25–29	811	9.8	5.8*	7.2*
	30–34	1,140	12.4	5.8*	9.3*
	35–39	1,315	14.5	7.5*	9.2*
	40–44	1,335	15.8	5.0*	8.6*
	45–49	1,291	17.3	6.3*	13.8
	50–54	1,127	16.9	6.0*	11.0*
	55–59	960	16.0	6.2*	10.6*
	60–64	750	14.0	5.6*	9.1
	65–69	698	14.3	5.9*	11.0*
	70–74	558	13.0	6.7*	6.7*
	75–79	507	14.4	4.9*	12.5*
	80–84	252	10.1	2.6	11.3*
	85+	179	8.1	2.6*	2.6*
Histology^e^	Papillary	11,803	5.2	6.7*	11.5*
	Follicular	2,312	1.0	3.1*	5.5*
	Other	1,461	0.6	0.4	0.4
Male	Papillary	2,817	2.7	6.1*	9.7*
	Follicular	619	0.6	3.0*	3.0*
	Other	463	0.5	0.1	0.1
Female	Papillary	8,986	7.7	7.1*	12.0*
	Follicular	1,693	1.4	3.8*	6.0*
	Other	998	0.8	0.5	0.5
Stage^f^	Local	9,775	4.35	6.1*	12.8*
	Regional	3,476	1.54	10.2*	9.9*
	Distant	876	0.39	7.2*	4.5*
	Unknown	1,449	0.64	3.6*	−4.5*

^a^Data are from the New Jersey State Cancer Registry, New Jersey Department of Health and Senior Services, November 2008 analytic data file. All primary invasive cases of thyroid cancer diagnosed between 1979 and 2006 are included except death certificate only (116) and autopsy only (45) cases.

^b^Rates are age-adjusted to the 2000 U.S. Standard Population direct method, except rates by age, are age-specific. All rates are expressed as number of cases per 100,000 population. Age-specific rates were not calculated for men due to small numbers.

^c^AAPC: Average Annual Percent Change, calculated from the National Cancer Institute's Joinpoint software.

^d^API is Asian and Pacific Islander; the API data are for 1997–2006 only. The ethnicity data are for 1997–2006 only, as complete ethnicity data are not available before 1990. Race and ethnicity are not mutually exclusive; the white and black race categories include white and black Hispanics.

^e^ICD-O-3 Codes for papillary are 8050, 8052, 8130, 8260, 8340–8344, 8350, 8450, 8452 and for follicular are 8290, 8330–8332, 8335.

^f^The 1979–2006 AAPCs should be interpreted with caution as the percentage of unknown stage was over 10% for most years 1979–1995.

*Statistically significant: the 95% confidence interval does not include 0.

**Table:** (a) Female thyroid cancer incidence rates by birth cohort, year of diagnosis, and age, New Jersey, 1979–2006, *N* = 11, 215^†^.

Year of diagnosis	Age at diagnosis													Mid-year of birth cohort^‡^
	20–24	25–29	30–34	35–39	40–44	45–49	50–54	55–59	60–64	65–69	70–74	75–79	80–84	
													9.0	1899
												9.4	8.4	1904
											5.8	9.9	8.5	1909
										7.7	8.1	9.5	6.4	1914
									7.1	10.2	9.2	10.7	11.8	1919
								7.8	8.2	10.9	13.3	20.5	18.2	1924
							7.7	8.1	10.2	12.3	18.5	30.2		1929
						6.8	10.0	10.2	11.4	22.7	29.4			1934
					8.5	9.4	10.6	12.5	23.8	30.2				1939
				6.6	9.9	11.1	12.9	25.6	29.7					1944
			6.6	9.4	9.2	11.7	24.2	34.1						1949
		5.1	8.2	9.5	13.8	25.9	36.1							1954
1979–83	4.1	6.7	8.4	13.1	24.5	36.9								1959
1984–88	4.8	8.9	12.1	21.7	27.4									1964
1989–93	5.1	9.3	19.0	29.8										1969
1994–98	6.0	15.7	27.1											1974
1999–03	9.0	19.0												1979
2004–06	10.6													1984

^†^Data are from the New Jersey State Cancer Registry, New Jersey Department of Health and Senior Services, November, 2008 analytic file. All primary invasive cases of thyroid cancer diagnosed between 1979 and 2006 are included except death certificate only and autopsy only cases. Cases  0–19 and 85 or older were excluded (*n* = 462). Rates are number of cases per 100,000 population and are not age-adjusted.

^‡^Birth cohorts are nine-year overlapping groups labeled by the mid-year, for example, the 1899 birth cohort includes people born in 1895–1903.

**Table:** (b) Male thyroid cancer incidence rates by birth cohort, year of diagnosis, and age, New Jersey, 1979–2006, *N* = 3, 772^†^.

Year of diagnosis	Age at diagnosis													Mid-year of birth cohort^‡^
	20–24	25–29	30–34	35–39	40–44	45–49	50–54	55–59	60–64	65–69	70–74	75–79	80–84	

													5.4	1899
												3.9	5.4	1904
											3.3	7.7	5.0	1909
										6.2	5.5	6.0	6.5	1914
									3.4	5.2	6.9	5.6	8.9	1919
								4.0	5.7	6.9	7.2	12.8	11.3	1924
							2.8	5.4	6.1	7.5	16.0	16.7		1929
						2.8	4.4	5.8	8.5	13.0	15.4			1934
					2.9	4.7	4.5	6.5	10.0	17.1				1939
				2.5	2.9	4.0	5.9	12.1	14.0					1944
			2.2	3.5	4.5	5.9	9.1	14.5						1949
		1.2	2.0	2.3	3.5	7.1	11.3							1954
1979–83	0.9	1.8	2.1	3.3	6.4	9.4								1959
1984–88	0.9	2.1	3.3	5.3	7.9									1964
1989–93	1.0	2.0	3.3	6.8										1969
1994–98	1.9	3.4	6.1											1974
1999–03	1.8	3.1												1979
2004–06	2.6													1984

^†^Data are from the New Jersey State Cancer Registry, New Jersey Department of Health and Senior Services, November 2008 analytic file. All primary invasive cases of thyroid cancer diagnosed between 1979 and 2006 are included except death certificate only and autopsy only cases. Cases  0–19 and 85 or older were excluded (*n* = 127). Rates are number of cases per 100,000 population and are not age-adjusted.

‡Birth cohorts are nine-year overlapping groups labeled by the mid-year, for example, the 1899 birth cohort includes people born in 1895–1903.

**Table 3:** Thyroid cancer incidence rates and rate ratios by socioeconomic status (SES) category, New Jersey, 1998–2002, *N* = 3, 924^a^.

SES category^b^	Total			Male			Female		
	Cases	Rate^c^	Rate ratio^d^	Cases	Rate^c^	Rate ratio^d^	Cases	Rate^c^	Rate ratio^d^
Category 1	1,281	10.6		324	5.5		957	15.4	
Category 2	1,185	10.2	1.0	300	5.5	1.0	885	14.7	1.0
Category 3	898	8.3	0.8*	225	4.5	0.8*	673	12.0	0.8*
Category 4	560	6.5	0.6*	116	3.0	0.5*	444	9.7	0.6*

^a^Data are from the New Jersey State Cancer Registry, New Jersey Department of Health and Senior Services, November 2008 analytic file. All primary invasive cases of thyroid cancer diagnosed between 1998 and 2002 are included except cases identified only through death certificates or autopsy reports and cases not geocoded to a census tract (*n* = 51).

^b^Census tracts were grouped into SES quartiles using the deprivation index scores. The deprivation index was developed with New Jersey 2000 U.S. Census data using principle component analysis. Census tract deprivation index scores in Category 1 were –2.23 to –0.71, in Category 2 were –0.72 to –0.14, in Category 3 were –0.13 to 0.59, and in Category 4 were 0.60 to 0.32. Category 1 is the highest SES category and Category 4 the lowest SES category.

^c^Rates are age-adjusted to the 2000 U.S. Standard Population, direct method, and are expressed as the number of cases per 100,000 population.

^d^The rate ratio is the rate for this SES category divided by the rate for SES Category 1 (referent rate).

*The rate for this SES category is significantly lower than the rate for SES Category 1, *P* < 0.05.
